# Retraction: Increased Six1 expression in macrophages promotes hepatocellular carcinoma growth and invasion by regulating MMP‐9

**DOI:** 10.1111/jcmm.18249

**Published:** 2024-03-20

## Abstract

Yongyu Zhang, Shiji Wang, Zhongmin Liu, Lewei Yang, Jian Liu, Ming Xiu. Increased Six1 expression in macrophages promotes hepatocellular carcinoma growth and invasion by regulating MMP‐9. J Cell Mol Med. 2019; 23: 4523–4533. https://doi.org/10.1111/jcmm.14342.

The above article, published online on 02 May 2019 in Wiley Online Library (wileyonlinelibrary.com), has been retracted by agreement between the authors, the journal Editor‐in‐Chief, Stefan Constantinescu, The Foundation for Cellular and Molecular Medicine and John Wiley and Sons Ltd. The authors asked to retract the article as they found that the experiments presented were not reproducible, due to a possible cell line contamination, and therefore the conclusions of the paper are not supported. Subsequently, an investigation into concerns raised by a third party revealed inappropriate duplication of images within the article (Figure 2C,D subpanels) as well as between this (Figure 2B subpanels) and four other articles that were previously published in a different scientific context. Thus, the editors consider the conclusions of this manuscript substantially compromised. Although the authors initially asked for the retraction, when asked to approve the retraction wording, they remained unresponsive.

Yongyu Zhang, Shiji Wang, Zhongmin Liu, Lewei Yang, Jian Liu, Ming Xiu. Increased Six1 expression in macrophages promotes hepatocellular carcinoma growth and invasion by regulating MMP‐9. J Cell Mol Med. 2019; 23: 4523–4533. https://doi.org/10.1111/jcmm.14342.

The above article, published online on 02 May 2019 in Wiley Online Library (wileyonlinelibrary.com), has been retracted by agreement between the authors, the journal Editor‐in‐Chief, Stefan Constantinescu, The Foundation for Cellular and Molecular Medicine and John Wiley and Sons Ltd. The authors asked to retract the article as they found that the experiments presented were not reproducible, due to a possible cell line contamination, and therefore the conclusions of the paper are not supported. Subsequently, an investigation into concerns raised by a third party revealed inappropriate duplication of images within the article (Figure 2C,D subpanels) as well as between this (Figure 2B subpanels) and four other articles that were previously published in a different scientific context. Thus, the editors consider the conclusions of this manuscript substantially compromised. Although the authors initially asked for the retraction, when asked to approve the retraction wording, they remained unresponsive.

